# Mentoring: A Traditional Cook Island Approach to Support Men

**DOI:** 10.1177/0306624X231172638

**Published:** 2023-05-13

**Authors:** Tess Patterson, Richard Egan, Julien Gross, Jessica Leov, Linda Hobbs, David La Rooy

**Affiliations:** 1 University of Otago, Dunedin, New Zealand; 2 North-West University, Potchefstroom, South Africa; 3 University of Waikato, Hamilton, Waikato, New Zealand

**Keywords:** men mentoring, rehabilitation male offenders, unique cultural approach, pacific approach, peer mentor

## Abstract

The present study examines a unique Cook Island approach to the rehabilitation and support of men, particularly those who have been convicted of criminal offending or who are experiencing other mental health or interpersonal difficulties. The culturally appropriate method of enabling change is offered via a community-based 24-hr mentoring system to support men. Run by men, the program is based on traditional Pacific ways of male mentoring in which one man helps another. This study examines the male mentoring program via qualitative analyses of semi-structured interviews. Seven men who had experienced mentoring and six mentors who deliver the program describe the mentoring system and their experiences. The study identifies several perceived benefits or themes in relation to the program. The unique Cook Islands’ male mentoring program is viewed as beneficial in that it allows males to be open and supported to make change to be re-absorbed into the community, have healthy functioning, and reduce re-offending via the ongoing supportive care.

## Introduction

Domestic violence is a serious issue in the Pacific region with recent estimates indicating that one third of woman in the Cook Islands have experienced some type of domestic violence (Heard et al., 2020; Te Marae Ora, Ministry of Health Cook Islands, 2014). The Pacific Prevention of Domestic Violence Program (PPDVP), an initiative of the New Zealand Agency for International Development (NZAID), New Zealand Police and the Pacific Islands Chiefs of Police, is a key player working in the prevention of domestic violence in the Pacific region. The Cook Islands’ PPDVP In-Country Report (Kingi & Roguski, 2011), highlights that although there is an increased awareness and reporting of domestic violence as a criminal matter in the Cook Islands, there is also a lack of intervention and treatment program options for those who commit domestic violence. Also to consider, although there are a number of therapeutic interventions that are provided for men who engage in domestic violence (Foote et al., 2014) most of these approaches have limited effect in reducing or stopping domestic violence (Day et al., 2009). Furthermore, these interventions are largely based on Western philosophies that do not necessarily consider the importance of cultural suitability and context (Brown & Ross, 2010; Farrall, 2004; Foote et al., 2014; Haney & Zimbardo, 1998; Mitaera, et al., 2018) and the effectiveness of Western interventions in Pacific countries is thus unknown (Haldane, 2009). There is, therefore, a pressing need to examine alternative methods of domestic violence intervention that are culturally appropriate and are effective in facilitating real change for those who perpetrate domestic violence.

One possible alternate intervention involves employing the use of peer-support or “mentoring” to improve outcomes for men involved in domestic violence. The success of mentoring and/or peer-support programs has been the subject of several recent literature reviews that have shown reductions in reoffending in the prison service (Buck et al., 2022), and reduced inpatient use and improved recovery outcomes for people with mental illness and/or substance abuse problems (Chinman et al., 2014). However, other commentators have noted mixed results on reductions in reoffending with some studies demonstrating a reduction in reoffending whilst others did not (Jolliffee & Farrington, 2008). There are also meta-analyses that have examined the effectiveness of mentoring and peer-support programs more generally and in relation to mental health. These also have produced contradictory results. For example, Pfeiffer et al. (2011) examined studies that used randomized control trial methodology and found that peer-support reduced the symptoms of depression, whereas Lloyd-Evans et al. (2014) found that peer-support had no effect on mental health symptoms. Notwithstanding that studies to date have been conducted within a western cultural context, additional limitations of these studies are that they were largely quantitative in nature and focused on narrow ranges of outcomes which may go some way to explaining the mixed results.

Given that studies to date have not considered peer-support or mentoring in a Pacific cultural context the current study employed a qualitative research methodology that offered an opportunity to look at a mentoring program running currently in the Cook Islands with a view to exploring more broadly and in-depth the potential benefits. The Cook Islands’ male mentoring program (the “mentoring program”) provides a service for men who have committed domestic violence offences or other criminal behavior, been imprisoned or are experiencing mental health or interpersonal issues. Specifically, the mentoring program offers a community-based system to support men with their difficulties, to facilitate change in behavior and to ultimately prevent re-offending. Their mentoring approach is based on traditional Pacific cultural ways and involves one to one peer mentoring, with an older man (the “mentor”) mentoring a younger man (the “client”). This treatment modality is not available in New Zealand and is unique to the Cook Islands. The Cook Islands’ male mentoring program offers us an opportunity to examine the effectiveness and benefits of this therapeutic modality and to consider its application to New Zealand and other Pacific countries. Using a qualitative approach, the current study aimed to evaluate the dynamics of the mentoring program by exploring themes of the impact of the program and how it is viewed to be of value or may assist in behavior change.

## Method

Ethical approval was granted by the University of Otago Ethics Committee (ref 14/191) and a permit to undertake research in the Cook Islands was granted to the first author by the Foundation for National Research, Office of the Prime Minister, Rarotonga. Support for the research project was given by the Pacific Prevention of Domestic Violence Program (PPDVP), New Zealand Agency for International Development (NZAID), New Zealand Police and the leaders of the Cook Islands’ male mentoring program.

### Participants

The participants consisted of both mentors and clients of the Cook Islands’ male mentoring program that was available on the main island of Rarotonga. Six male mentors were interviewed who ranged in age from 53 to 77 years old. All mentors identified as Cook Island Māori. Three of the mentors had seen two or more clients while the remaining three had yet to mentor an individual client. All mentors interviewed had been selected and trained by peer support elders and were fully prepared and informed in relation to peer supporting clients.

Seven male clients of the mentoring program were also interviewed. The male clients ranged in age from 35 to 48 years old (two clients did not provide their age). One client identified as European while the remaining six were Cook Island Māori. Four clients were receiving mentoring in relation to their prison sentences, two were receiving mentoring for stress and mental health issues, and one was receiving mentoring for anger and health issues. There was significant overlap in reasons for clients seeking mentoring.

### The Cook Islands’ male mentoring program

The Cook Islands’ male mentoring program is a unique treatment intervention service for men who have committed domestic violence offences, been imprisoned or are experiencing other personal or mental health difficulties. The 24-hr community-based service is offered to support men in a number of ways (e.g., crisis intervention, provision of material needs, counseling and support) and to prevent those who have offended from re-offending. The program is based on traditional Cook Island cultural ways, involves one-to-one peer mentoring and is unique to the Cook Islands. Referral processes are from governmental and non-governmental agencies, as well as self-referral.

### Procedure

Past and present mentors and clients were invited to take part in the study if they have been involved in the mentoring program within recent years. They were provided information about the aims of the study and asked to indicate whether they would like more information about the study and/or whether they would like to participate. A Cook Island Research Assistant provided this information, answered questions, and obtained informed consent.

Semi-structured interviews were conducted with the participants on Rarotonga, Cook Islands by one of two interviewers, a male Cook Island Research Assistant completed interviews with four clients while a male New Zealand European interviewer (second author, RE) completed nine interviews: six with mentors and three with clients. The semi-structured interview explored mentors’ and clients’ experiences within the mentoring program. Participants were asked questions that included what the mentoring involved for them personally, the perceived benefits and limitations of the program, and how the mentoring program related to the Cook Islands’ culture and religion. Interviews were recorded and then transcribed verbatim. To protect the anonymity of individuals and organizations each participant was assigned a research number and other identifying material was removed from the interview transcripts.

### Analysis

Interpretative Phenomenological Analysis (IPA) was used to explore participants’ experiences within the mentoring program. IPA does not seek to test a hypothesis but instead is an inductive approach that requires a detailed exploration of each participant’s reported experiences (Braun & Clarke, 2006; Smith & Osborn, 2015). We adopted the IPA approach because it is a bottom-up process that allows the researcher to develop themes from the data rather than fitting the data into pre-existing themes. By using this approach, the analysis is not a discrete step, but instead, an iterative process that involves multiple readings and immersion in the text to confirm that the emergent themes and interpretations are supported by the data. Successful IPA also requires interpretation in which the analyst must enter the research process therefore IPA needs to be recognized as subjective process in which the interviewer interprets each participant’s account through the lens of their own attitudes and experiences (Larkin et al., 2006; Smith & Osborn, 2015). This process means that the results are not stand-alone facts but are instead a result of the interaction between the interviewer, interviewee and the researchers who undertook the IPA process (Reid et al., 2005).

The first stage of the analysis was familiarization with the data. The transcribed interview was read through once to get a general understanding of the experiences of each participant. After this initial reading the interview was then read through twice more and annotated by the researcher with their initial impressions of the interview. Annotations identified interesting or important concepts that were present in the interview. There was no specification as to what had to be commented on.

After the initial annotations were completed, the transcript was read again, and emerging themes were identified with initial annotations expanded on and developed to give a more coherent explanation of the emerging themes. NVivo, a qualitative data analysis software package, was used to assist with the identification and classification of themes. Potential themes were created as nodes using the NVivo software. Potential quotes to support these emerging themes were also highlighted and organized under each of emerging nodes. This process was followed for each interview and allowed the researcher to develop ideas about consistencies and emerging themes across the interviews. Analysis of later interviews was informed by the analysis of earlier interviews.

Connections and similarities between the themes across the interviews were identified and the themes were ordered into specific patterns and clusters of meaning. Themes were then refined and reordered. Some of the themes were closely linked and could be combined. Other themes that had originally been considered as main themes were reordered to subthemes and broader themes were created.

The final step of the analysis was consolidation and summarization. This involved taking the identified themes and turning them into a narrative account which encapsulated the participants’ lived experiences. The clients and mentors who participated in the study were invited to a workshop in Rarotonga where the thematic analysis was presented and they were provided with an opportunity to provide feedback on the themes via participatory discussion. Feedback from this workshop was that the themes identified resonated with the members of the Cook Islands’ male mentoring program and no changes were made to the identified themes.

The narratives explored the identified themes and are accompanied by verbatim quotes from the interviewees to illustrate and support the choice of themes. Quotes have been included from all participants. To ensure confidentially of identity and to ensure quotes from the same individual cannot be linked we have chosen to not include pseudonyms to identify the participant who made each quote. Quotes are identified as being from a Client or a Mentor.

## Results

The analysis identified four interrelated themes comprising: (1) gender and masculinity, (2) Cook Island cultural identity, (3) religious spirituality, and (4) shared experiences. These themes inform and influence the mentoring experience of both the clients as well as the mentors. Although the participants had a diverse range of experiences, these themes highlight common factors within the Cook Islands’ male mentoring program. While distinct, none of the themes exist in isolation and they are somewhat interrelated. References to “the men” throughout refer specifically to participants and are not a generalization to all Cook Island men. Participants’ experiences are discussed within each of the four themes with supporting quotes from the interviews.

### Gender and Masculinity

The Cook Islands’ male mentoring program is run by men for men. The theme of gender and masculinity incorporates how the client’s masculine identity influences their behavior, and the role that gender plays in the mentoring program. A reoccurring theme throughout the interviews were representations of masculinity, and how this in turn influences men’s ability to discuss their problems. Men felt that they were expected to conform to specific patriarchal ideals, most notably as the financial providers for their family, appear strong, and emotionally restrained. These restrictive representations of masculinity lead to men being afraid to seek help because they feel embarrassed about their situation and their perceived weakness. This also created a barrier for men to discuss and attempt to deal with their problems. Clients stressed the need for greater awareness of the mentoring program due to men’s unwillingness to speak out about their problems and suffer in silence.

Client: *I seeked (sic) help, because most men don’t, they don’t seek help, they keep it in, they try sort it out, they try to be the ‘man’, you know.*

A mentor, who had at the time of the interviews had mentored the most clients, stated that the desire to talk to another man about their problems was, in fact, a common occurrence. Generally, the mentors were aware of the uniqueness of the mentoring program and the unique opportunity it provides for men to talk openly about their problems with other men. This gendered approach of men working with men is an aspect of the program that appeals to clients and helps them benefit from the program.

Mentor: *Yeah, I, ah I think, I think for most clients. . .this thinking back to my old clients the most common remark has been, ‘I really need another man to talk to’ you know, somebody who understands where I’m coming from, that, that’s pretty much been the common thing you know that I get from the client. It’s that man to man, um yeah and just knowing that, that you are there for them.*

The importance of talking to a man is also reflected in the interviews with the clients. Clients feel that, when speaking to another man, they are better understood and in turn will be more frank and therefore better able to deal with underlying problems. The ability to speak to another man who has shared experiences means men can openly express problems free of judgment.

Client: *People are looking for help, there is no place to go, if I am a man I’d rather go to a man, yeah. . .Sometimes of course it’s hard for me to tell my problem to a lady. . .Yeah, those are the sort of things we need to look at so at the [Cook Islands’ male mentoring program] the man will go there. . .So we deal with their issues from a man to a man (perspective) and it works and they will open up, they will.*

Client: *. . . how would a woman’s mind think about, you know, helping the men out, with their problems.*

There is also the belief that men might not be as open or forthcoming when speaking to women from fear of judgment or misunderstanding.

Mentor: *it’s better to refer to us the men [the Cook Islands’ male mentoring program] so that we can speak to them freely because we’ve got nothing to hide, eh?. . . He [the client] talks to the women and he [the client] can’t tell them everything.*

Some participants were also willing to openly share their personal views surrounding women and domestic violence. Problematic beliefs included the idea that women were responsible for the abuse they received, and that physical violence was an acceptable response to certain situations.

Client: *I’m not too sure but I think, well um, half of the domestic violence is fuelled by the women anyway.*

Overall, this theme demonstrates the challenges that men feel around their masculine identity and how important it is that the mentoring program provides men with the opportunity to speak to another man. The mentoring program acknowledges that sometimes it is hard to be a good man and mentors offer to walk shoulder to shoulder with their clients providing gentle guidance and support (both traditional and religious) while men negotiate what it means to be a man in a modern Cook Island society.

### Cook Island Cultural Identity

The Cook Islands’ male mentoring program is a system founded on traditional Cook Island beliefs of mentoring. The theme of Cook Island cultural identity incorporates the affirmation of and the potential loss of a Cook Island identity for clients. This theme explores how the mentoring program fits with traditional Cook Island beliefs and practices.

The Cook Islands are undergoing dynamic changes and with increasing westernization, traditional ways of life are changing. With this change comes a challenge to Cook Island cultural identity. Westernization and progress are not necessarily negative; however, some participants express difficulty maintaining a unique Cook Island self within a changing society.

Client: *That’s it um, our society is changing, you know, pretty well that um I like to say this, um, our way of living is no longer our way. It is more western, yeah, so that’s why it’s changing, that’s how I see it.*

The New Zealand—Cook Island relationship was considered by some participants. While New Zealand presents viable economic opportunities for men on the island, the relationship does come with consequences. Except for two individuals all participants had lived and worked in New Zealand for an extended period of time. One mentor spoke as to how this could further erode cultural identity:

Mentor: *and we don’t have the leaders in New Zealand. Not like the Samoans. You see the [Famous Samoan named] they’re in the face of their young people all the time . . .and their young people know how to speak the language. . .*

This mentor also talks about the loss of the Cook Islands’ Māori language and how this further distances Cook Islanders from their heritage.

Mentor: *I’m brought up in New Zealand. I had to re-learn my language. . .and, and I’m still doing it, you know and, and, my children do not know it, my grandchildren do not know it . . .So that’s, that’s a problem that we having. . .So even here. We, we roped into the westernized influence ah, I’m talking about the English language and so that, that [English] is their first language over here (in The Cook Islands).*

Mentor: *That’s [English] their first language. There’s only one school [in the Cook Islands] that is ah, that curriculum is veered towards speaking [Cook Island Māori] but even the children there speak more English than Māori so there’s most of our children speaking English at school. It’s terrible.*

One client spoke about how a lot of the Cook Islands’ cultural and historical knowledge is more readily accessible in New Zealand or Australia compared to in the Cook Islands.

Client: *Put them on their journey to their culture. There are some of us who want to revive the culture or research the history and all that but you can’t do it here [in the Cook Islands]. All the information you need is all in New Zealand, England, or Australia.*

This comment highlights the disconnection that the client feels with his own cultural identity. Despite living on Rarotonga, the client feels that he needs to travel to New Zealand to fully explore his cultural heritage. Increasing immigration and tourism also lead to some interviewees expressing being marginalized and economically disadvantaged in their own country. They expressed feeling disillusioned with negative stereotypes of being a native Cook Islander, the idea that locals were lazy and uneducated. A mentor described that this loss of identity was why men were struggling because they did not have a sense of who they were.

Mentor: *I believe that’s why our Cook Island men in New Zealand, as are most of the Pacific nations, are having difficulties. It is an identity problem.*

The mentoring program seeks to revive established Cook Island traditions of mentoring. Conversations with the participants highlight the notion of a community raising the individuals. Participants talk about how when an individual needs direction Cook Island tradition would dictate that he is sent to the male elders (Papas) of the community for guidance or reprimand.

The mentoring approach is grounded in Cook Island philosophies of support and community. In keeping with Cook Island tradition, men can talk openly with other men as they have shared experiences and develop understanding and caring relationships. Below is an excerpt from the founding mentor explaining how the mentoring program is grounded in Cook Island culture.

Mentor: *So it was really born out of our, a man walking with another man, holding another man’s hand which is predominantly a, ah, ah a Pacific not just a [Cook Island] but a Pacific culture where you know . . .there’s, you’re, you are looking at two men who are best friends, who trust each other, who open to each other, and more likely to receive rebuke from each other rather than from somebody else.*

The western concept of mentoring was already an inherent part of the Cook Island culture that has been partially lost through modernization and colonization. Participants see the mentoring approach as an authentic part of Cook Island culture and found defining it within the Cook Island culture is difficult because, as one mentor said: *“. . .it’s in our blood.”*

### Religious Spirituality

The theme of religious spirituality incorporates the role that religion and faith has within the mentoring program. There is a strong religious component to the mentoring service. Many of the mentors are pastors or have a connection to the one of the Churches on the island. Three of the mentors interviewed were ordained pastors. The mentors’ faith frequently informed their interactions with the clients and conversations often centered on religion; and scripture may be used to provide guidance to the men.

The mentors acknowledge that the Church plays an influential role in Cook Island men’s lives:

Mentor: *. . . most of the men have been bought up in the church. Whether they are catholic or ah they are Cook Island Church.*

Mentor: *and so I think Christian [Christianity] and religion plays a very vital role in shaping the way they think. . .and the Church has a big influence on our men here.*

One Cook Island mentor, in conversation with the interviewer, explains how Christian teachings about how to be a man can be misinterpreted.

Mentor: *I found that some of the teachings [of the church] have been ah, have formulated within their minds that this is what a man is, when he becomes a man, I’m the man, you know, you do what I say, or I’ll clip your ears you know.*

Mentor: *and they, so what I see is that they kind of, lording it over their wives instead of, which comes from a, a, an incorrect picture of what the scripture is saying.*

This mentor goes on to provide an example of how he reframes some of the scriptures for men, for example, in relation to St Paul he notes:

Mentor: *So, you have a problem with your wife, ‘yes, she’s not submitting to me’, they both go to church you know . . . Okay, so, what do you understand by that, the submission to the husband? ‘She should be doing what I say, if I tell her to do something she should be doing it’ . . . I said okay, let me say, are you aware of the first part of the scripture? He goes ow ‘what do you mean?’ There’s the first part of the scripture it says the husband submits himself to God and he goes ‘what does that mean?’ . . . I say it simply means this. When you are submitted to Christ you won’t have any problems with your wife but if you’re not submitted to Christ in one area you’ve got a problem.. . . . . . That’s what that passage is all about because the submission is not from you [as the man], it’s from Christ. That’s the submission, it comes through you and through your wife and so if it breaks down from where you are, expect a revelation . . . and they go, oh, ‘I didn’t realise it was like that’ and I said you go away and think about it. Is there just one area where you are not fully submitted to Christ? He says ‘ow I know one already’ [laughs] I say, I figured that, so first of all you have to deal with your own issues, if you’re going to expect you know submission from your wife, and that’s not lording it over your wife, your wife is supposed to be walking next to you in the sun, not at the back of you. I find that I had to do a lot of that [reframing].*

Clients generally appear to appreciate this religious spiritual approach and for many it is a central part of their mentoring experience. In fact, one client attends the mentoring primarily for religious spiritual support.

Client: *Besides the spiritual thing, that’s why they need the support eh? That way they can broaden their horizon. Basically, I come here for spiritual counselling*.

Religious scripture and morals help frame the way men were approaching their problems and can be used as a coping mechanism.

Client: *Well they, they, um, they bring service [religious service], you know the service and they gave me something, just to help me and also with the um, the word of God to help me with the situation, calm down my, you know . . . and the other thing, the mentor always talk about is um, um, he talks to me about spiritual things and he talks to me about love which I like and I um, link with that, yeah and I always believe that while you have gone through a rough time, only you can change.*

Many clients experienced a reconnection with their faith and their religion. Having been raised with the teachings of the Christian church, some clients found the religious guidance comforting.

Client: *Yeah, I felt that he was very understanding, and ah he, he . . . um I was happy that he was a Christian person, so he referred more to the Bible. Because that was another thing, that caused me imbalance, because I had not been to church for a while and I tend to forget, you know, simple promises that God has promised. You know, for example, ‘cast all your cares upon me, for he careth for you’. Just those text you know, I had forgotten, and those few years they slip you know, and he talked about the Bible, and he talked about the Ministry, and I thought, this is me sitting there and I said ‘oh yeah I remember that, I remember that’. So that helped me . . . to remind me, that that’s what I was.*

The men also mention how institutional faith is often rigid or lacked a level of understanding about their situation. The spiritual guidance of the mentoring program was more flexible. Discussions of faith are personal and related to the client’s experience. Mentors did not shy from addressing difficult topics (i.e., violence, alcoholism) which some clients felt the church would often neglect. Clients found the spiritual discourse of mentoring sessions was more personalized and offered them an opportunity to explore their faith in a way that suited them.

Client: *Somewhat, not 100%, it’s [church] is not like the mentoring. It’s different from the mentoring because the church is about love and compassion and the Bible and so forth, which is all good, but it’s [mentoring] is not all like the power. Like [mentor name] he really was lying in the ditch and having nobody and then dragged himself out, so [mentor name] went through the bad things and many people in church just didn’t, you know.*

Some mentors and clients took a broad spiritual approach, while others found solace in the more fundamental Christian scripture. Religion and spirituality play a significant role in men’s mentoring experiences and is a core component of many men’s experience.

### Shared Experiences

This theme explores the role that shared experiences plays in aiding the mentor relationship and how trust and respect are core components of the mentor relationship. The diverse experiences and age of the mentors aid their relationship with clients. Mentors often have first-hand knowledge of the challenges the client face be it with addiction, offending and/or financial stress.

Mentor: *the mentor is another person, another individual that’s within the circle and so they become your peer and you know there is like in a classroom at school, peer students and then the other students. Ours goes beyond that. So these, we, we provide the mentors that are a lot more experienced, a lot more older, ones that may have been in the same circle at some time; so in that sense I guess you can still use the word peer because there is a, a relationship that has already been there. For a time they have been in prison which is the case for a lot of our clients.*

Clients appreciate this and often comment on how this helps their relationship. One client was of European descent however this did not hinder his relationship with his Cook Island mentor because they had the shared experience of problematic alcohol abuse. This shared experience helped the client connect and be honest with his mentor because he felt he understood what he was going through. In conversation with the interviewer this client explains this relationship with his mentor:

Client: *mmhhm, has to do with his personality you know he is very empathetic, very experienced . . . he had like a very heavy drinking problem himself where it got out of control;*

Client: *He, he um, he was um, you know at one point we joked and um I said to him ‘You know but with me it got so excessive, it got so bad you know you give me a fatty cheeseburger to eat and I will drink anybody on this island under the table’ . . .*

Client: *We clicked right away, it was no problem.*

Clients felt that mentors with shared experiences had a deeper understanding of what they were experiencing which allowed clients to open-up and talk honestly about their experiences.

Client: *Well, if they do come from a background like that, they can understand what the actual client is going through. If they used be a violent person or they used to consume a lot of alcohol or they used to maybe they were rough when they were teenagers you know. I reckon every mentor should have a background like that because how can you communicate well with someone if you’ve never done anything that they’ve done?*

Client: *[speaking in relation to his mentor] . . . I could relate to him and talk to him ‘cause, I don’t know if these other guys have ever been bad or anything.*

Mentor: *He [the mentor] can says ‘I’ve been to prison, I know where you’re coming from.’ For a lot of others, the counsellor who is working with client, say’s ‘I understand’ but he’s [the counsellor] never been in prison.*

Shared experiences seemed to play a significant role in developing a strong mentor—client relationship. This builds on the ideas discussed in the previous themes of gender, Cook Island cultural identity and spirituality; those commonalities, be they gender, experiences or faith, are important building blocks for clients and their mentors’ rapport. Evident throughout the interviews with the clients is their love and appreciation for their mentor. Clients express gratitude for their mentors which is reflected in the comments below. They trust the guidance that their mentor offered.

Client: *and it’s really been a big help, and I want to thank that person [peer mentor], yeah we did that and we came in and the [Cook Islands’ male mentoring groups] started to pop up from within prison and then it started to come outside [of prison], I lived with that and then when I came out [of prison] I still stayed [with the peer mentoring].*

Client: *I saw some people say ‘Oh I need to come to my saviour’ [laughs] that’s how this person put it eh? This is the guy [peer mentor] who is going to save me.*

Client: *well that’s how I see it? This is how I see these mentors because they spend their time um, understanding your issues with love to you.*

Client: *Very comfortable, the mentor is not there to judge, to criticize, to remind you, they’re there just to listen and understand about your problem that you are going through.*

## Discussion

Worldwide, the social, emotional and financial costs of violent or other offending are significant (McCollister et al., 2010). Successful rehabilitation of violent or other criminal offenders is important in relation to community safety and to stop further potentially traumatizing events occurring. Rehabilitative programs of criminal offenders vary and are largely based on Western philosophies that do not acknowledge the importance of the social and cultural context within which the programs are intended to operate (Brown & Ross, 2010, Farrall, 2004; Haney & Zimbardo, 1998). This study was the first to closely examine the mentoring of men in a particular Pacific cultural context and included men who had been in prison for assaultive events. By examining the Cook Islands’ male mentoring program in Rarotonga the study was able to identify the key themes that help understand the relationships between clients and mentors, and the importance of understanding clients’ identity and spirituality. The key themes identified in this research were: (1) gender and masculinity, (2) Cook Island cultural identity, (3) religious spirituality, and (4) shared experiences. The themes interacted uniquely in this cultural context.

The study supports the idea that men-talking-to-men in this cultural context facilitated meaningful communication and allowed clients to feel that they were understood given the overlap in shared experiences between the clients and mentors. Clearly, the included quotes illustrate that many sensitive topics are addressed through the client-mentor relationship that would have been otherwise difficult to discuss outside of this context. The clients clearly see the value in having this freedom, were confident that they were understood by their mentors, and were willing to listen to advice that helped change their behavior. For example, some of the men were able to express some highly problematic beliefs that included the idea that women were responsible for domestic violence. Expressing such problematic beliefs allows for the beliefs to be directly addressed and resolved. The struggle of defining masculinity is apparent in interviews with both the mentors and clients. In this fluid cultural climate men’s traditional roles and identities are being challenged and reshaped. This mentoring approach is intended to help increase the possibility that clients can have sensitive topics addressed, change their behaviors, and be re-absorbed or function in the community in a healthy and meaningful way. It also directly targets re-offending by providing support and guidance to reduce risk of re-offending. This study highlights the benefits of taking a cultural gendered approach to mentoring programs, and in particular, those designed to address serious wrongdoing.

Religious spirituality and religious belief were an important component of the mentoring program and many of the mentors were pastors or closely connected to the church. The mentors were able to identify the deeper role that religious spirituality played in supporting some of the men’s problematic beliefs. Some men believed that the scriptures placed the man at the center of family life—above the woman. The mentors found that as patriarchal and misogynistic views were elicited, the mentors were able to undermine these views using scripture, capitalizing on a perceived void in which men needed to redefine their identity of what it means to be a man. More broadly, some of the men were able to rebuild their lives by recognizing the importance of reconnecting with the church and their communities.

With respect to Cook Island cultural identity, some men noted the erosion of traditional values and practices; this loss was particularly felt in relation to language and being able to speak Cook Island Māori. The modernization and westernization of the Cook Islands, the need to work in New Zealand, and other major cultural changes may also directly contribute to crime and criminality (see Karstedt, 2001). Moreover, pressure to conform to patriarchal ideals, such as being financial providers for their family, with restricted economic opportunities, for example having to work abroad, may have left men more vulnerable and cut off from traditional support systems. Indeed, it was noted that the Cook Island Māori were not as well organized in terms of extending support to each other when working abroad (e.g., in New Zealand) as other Pacific cultures.

A limitation of this research was that the male participants were all members of the same peer-mentoring group and that the clients themselves were actively seeking support at the time of the study was conducted. Therefore, the perceived benefits of this program must be viewed as ones arising from men actively seeking to change their behavior (i.e., the findings of this study may not generalize to men who are not seeking change). Such willingness to seek professional help may have facilitated the men’s willingness to participate in the research and talk about their experience. That said, this Cook Island male mentoring rehabilitation program seemed to fill a gap in treatment for men genuinely willing to change their behaviors. There is a need, however, for follow up research (both quantitative and qualitative) to examine the long-term outcomes regarding the perceived value of this peer support mentoring in terms of changes in behavior or functioning, and any reduction in re-offending, particularly after peer support has been completed; based on the current finding we suggest that the rehabilitation treatment program could be replicated elsewhere and would be of benefit.

## Conclusion

The themes identified in this study are interconnected, but it is clear from the men’s stories that men-talking-to-men about their experiences, within this specific cultural context, allowed mentors to find rehabilitation pathways that were culturally relevant and ones that made sense to the clients. This approach served to remove barriers for men to talk about sensitive topics that they would otherwise not be able to. In the medium term, mentors saw the rehabilitation process as one where men were able to walk “hand-in-hand” along the rehabilitation pathway and that the men were not alone on that journey. Ultimately, by providing an alternate culturally relevant intervention (i.e., the peer support intervention) there is a hope to see reductions in re-offending rates and improved psychosocial functioning. With further research this culturally tailored intervention could serve as a treatment model to be used elsewhere in the Pacific.
